# Use of large scale EHR data to evaluate A1c utilization among sickle cell disease patients

**DOI:** 10.1186/s12911-021-01632-5

**Published:** 2021-09-18

**Authors:** Shivani Sivasankar, An-Lin Cheng, Ira M. Lubin, Kamani Lankachandra, Mark A. Hoffman

**Affiliations:** 1 School of Medicine, University of Missouri-Kansas City, Kansas City, MO USA; 2 Division of Laboratory Systems, Centers for Disease Control and Prevention, Atlanta, GA USA; 3 Centers for Disease Control and Prevention, Atlanta, GA USA; 4 Children’s Mercy Hospital, 2401 Gilham Road, Kansas City, MO 64108 USA

**Keywords:** Electronic health records, A1c, Sickle cell disease, Fructosamine

## Abstract

**Background:**

The glycated hemoglobin (A1c) test is not recommended for sickle cell disease (SCD) patients. We examine ordering patterns of diabetes-related tests for SCD patients to explore misutilization of tests among this underserved population.

**Methods:**

We used de-identified electronic health record (EHR) data in the Cerner Health Facts™ (HF) data warehouse to evaluate the frequency of A1c and fructosamine tests during 2010 to 2016, for 37,151 SCD patients from 393 healthcare facilities across the United States. After excluding facilities with no A1c data, we defined three groups of facilities based on the prevalence of SCD patients with A1c test(s): adherent facilities (no SCD patients with A1c test(s)), minor non-adherent facilities, major non-adherent facilities.

**Results:**

We determined that 11% of SCD patients (3927 patients) treated at 393 facilities in the US received orders for at least one A1c test. Of the 3927 SCD patients with an A1c test, only 89 patients (2.3%) received an order for a fructosamine test. At the minor non-adherent facilities, 5% of the SCD patients received an A1c test while 58% of the SCD patients at the least adherent facilities had at least one A1c test. Overall, the percent of A1c tests ordered for SCD patients between 2010 and 2016 remained similar.

**Conclusions:**

Inappropriate A1c test orders among a sickle cell population is a significant quality gap. Interventions to advance adoption of professional recommendations that advocate for alternate tests, such as fructosamine, can guide clinicians in test selection to reduce this quality gap are discussed. The informatics strategy used in this work can inform other largescale analyses of lab test utilization using de-identified EHR data.

## Background

Inappropriate test selection can lead to misdiagnosis, suboptimal treatment and unnecessary costs [1]. Patients with a genetic disease often require special considerations for test selection to screen and manage prevalent chronic diseases [2]. Sickle cell disease (SCD) is one of the most common severe monogenic hematological disorders worldwide [3]. SCD is a multi-system disease caused by mutations in the hemoglobin beta chain gene [4, 5]. In the US, SCD affects approximately 100,000 people, or one out of every 365 African-American births and one out of every 16,300 Hispanic-American births [6]. It has been estimated that 2.3% of the world’s population have sickle cell disorders [7]. In 2017, over 30 million people in the US (9.4%) and globally over 425 million individuals with type 2 diabetes mellitus [8, 9]. The standardized prevalence of type 2 diabetes mellitus among patients with SCD in the US showed a modest increase from 15.7% to 16.5% from 2009 to 2014 [10].

The glycated hemoglobin A1c test is the most commonly used laboratory test to assist in diagnosing and managing diabetes [11]. The A1c test measures glucose bound to the β-chain of the hemoglobin molecule [11]. In the presence of excess plasma glucose, the hemoglobin beta-chain becomes increasingly glycosylated, making A1c a useful index to monitor long term glycemic control in patients with diabetes mellitus [12]. The test measures the three-month average plasma glucose concentration, reflecting the average normal lifespan of red blood cells. A1c tests are not recommended for persons with SCD or other hemoglobinopathies and anemias in which the lifespan of red blood cells is shorter than the usual 110–120 days [13–15]. Three alternative tests provide accurate results when the red blood cell lifespan is shortened: fructosamine, glycated albumin, and 1,5 anhydroglucitol [16]. Of these, fructosamine has received considerable attention in the published literature [17–19]. The total concentration of fructosamine is predominantly a measure of glycated albumin and a minor contribution of other circulatory proteins such as glycated lipoproteins and glycated globulins [20]. The American Diabetes Association (ADA) advocates the use of alternative tests for patients with hemoglobinopathies because A1c testing can be unreliable [21]. This was also emphasized in guidance provided through a national information campaign of the National Institute of Diabetes and Digestive and Kidney Diseases in 2014 [22] and by the National Glycohemoglobin Standardization Program [23].

Adoption of the guidance to use an alternative test in lieu of A1c testing in SCD patients is poorly understood. Data derived from EHRs loaded into multi-institutional data warehouses provide a powerful resource for investigating these issues. These data warehouses provide a comprehensive and longitudinal collection of patient health care data and an emerging resource for health services analysis. Several studies have demonstrated that EHR systems can promote cost-effective and sustainable solutions for improving quality in medical care [24]. Multi-institutional data warehouses aggregating EHR data from multiple sites allow national level assessment, comparison of practices and analysis of outcomes across independent, non-affiliated, organizations to guide quality improvement initiatives and identify gaps [25]. One such data resource, Cerner Health Facts™ (HF), has been demonstrated to have frequency of diagnosis codes consistent with the HCUP National Inpatient Survey, indicating that multi-site EHR data warehouses can be representative of national trends [26].

We explore the trends in A1c testing among the SCD patient population in HF and evaluate whether facility characteristics affect these trends. The analysis also serves to establish a baseline that is important for assessing the effect of potential interventions to mitigate any quality gap.

## Methods

### Data source

This study used the de-identified HF data warehouse (Cerner Corporation, Kansas City, MO), which contains longitudinal patient data systematically extracted from the EHR at participating institutions and includes encounter data (emergency, outpatient, and inpatient), patient demographics (age, sex, and race), diagnoses and procedures, laboratory data, and facility characteristics. The HF release used for this work (2016) consisted of 386 million encounters, 4.3 billion lab results from 64 million patients, and other data from 863 US healthcare facilities. All admissions, inpatient medication orders and dispensing, laboratory orders, and specimens are date and time stamped, providing a temporal relationship between treatment patterns and clinical information. Consistent with HF policies, all data were de-identified in compliance with the Health Insurance Portability and Accountability Act (HIPAA) before being provided to the investigators. The facilities contributing data were each assigned a unique identification code. Longitudinal relationships between patient encounters within the same health system are preserved.

### Study cohort

We conducted a retrospective analysis of patients with a diagnostic code for SCD, including its variations, such as sickle cell thalassemia, using the International Classification of Diseases, Ninth Revision and Tenth Revision, Clinical Modification codes (ICD-9-CM and ICD-10-CM)*.* We excluded patients with a diagnostic code for sickle cell trait (ICD-9-CM: 282.5, ICD-10-CM: D57.3). The codes were selected based on clinical judgement and the Phenotype Knowledgebase (PheKB) [27]. PheKB standardizes machine-readable definitions of common diseases and provided a published algorithm to identify sickle cell disease cohort within EHRs using ICD-9-CM diagnosis codes. We accepted patients with a single encounter though other analyses have required two or more encounters with a SCD code [28]. The resulting definition groups (from ICD-9-CM codes) were combined with the appropriate ICD-10-CM codes to identify the sickle cell disease patient cohort (Table 1).

**Table 1:** The ICD-9 and ICD-10 CM codes used in this study to identify the Sickle cell population, grouped based on consensus definition from PheKB

Definition	ICD-9 CM codes	ICD-10 CM codes
Sickle cell thalassemia without crisis	282.41	D57.4 D57.40
Sickle cell thalassemia with crisis	282.42	D57.41 D57.411 D57.412 D57.419
HbSS disease unspecified	282.6 282.60	D57
HbSS disease without crisis	282.61	D57.1
HbSS disease with crisis	282.62	D57.0 D57.01 D57.02
Sickle cell/HbC disease without crisis	282.63	D57.20
Sickle cell/HbC disease with crisis	282.64	D57.2 D57.21 D57.211 D57.212 D57.219
Other sickle cell disease without crisis	282.68	D57.8 D57.80
Other sickle cell disease with crisis	282.69	D57.81 D57.811 D57.812 D57.819

### HF data extraction from patients diagnosed with SCD

This sickle cell-patient cohort was analyzed for A1c and fructosamine encounters based on the criteria that included patients, each of whom had at least one A1c test order (identified by LOINC codes: 55454-3, 41995-2, 4548-4, 17855-8, 4549-2, 17856-6), fructosamine test order (identified by LOINC codes: 33805-3, 15069-8, 53550-0), glycated albumin (identified by LOINC code: 13873-5, 1758-2) and/or 1,5 anhydroglucitol (identified by LOINC code: 53835-5) after the first use of a diagnosis code for sickle cell disease. We did not attempt to identify the purpose for the order (i.e., screening, diagnosis, or management) because any use of A1c testing is contraindicated for the sickle cell cohort. A1c and fructosamine encounters before 2010 were excluded from the analysis because the HF data architecture was updated in 2008–2009.

### Definitions and analysis of cohorts

An A1c encounter refers to a clinician interaction in which an A1c test is ordered. Testing trends are initially investigated by assessing the facility characteristics in the overall HF cohort and among three facility groups defined below to scan for facility level patterns that might corrrelate with test utilization. Patient characteristics within each facility cohort are assessed. If the date and time stamp of the A1c test were the same as that of the fructosamine test ordered for the specific patient, that A1c test was considered to be co-ordered with fructosamine.

We identified three facility groups (adherent, minor non-adherent and major non-adherent) from the study cohort based on the prevalence of the SCD patients having had A1c testing ordered. An adherent facility has documented evidence that they have patients with an SCD diagnosis code but not an associated A1c test code but also has data confirming that these facilities provide A1c testing to other patients. A non-adherent facility has at least one patient encounter with an SCD diagnosis code and at least one A1c test order for any SCD patient. We classified non-adherent institutions according to the percentage of SCD patients who received inappropriate A1c testing. We stratified the non-adherant institutions by quartile and focused our investigation on the first quartile (< 25th percent adherant) (NA-1) and the fourth quartile (> 75th percent non-adherant) (NA-4). We compared the three facility sub-cohorts (adherent, NA-1 and NA-4) to evaluate patterns related to test utilization at the facility and patient level.

Descriptive statistics were used to summarize the facilities and patients characteristics as mean (SD) or proportions. We used the R statistical software package (version 3.3.1) to perform Chi-square or Fisher’s exact test (sample size < 5) with the categorical data. Continuous data was evaluated using Student’s t-test (normally distributed) or the Mann–Whitney test.

## Results

### Study population

**Fig. 1:**
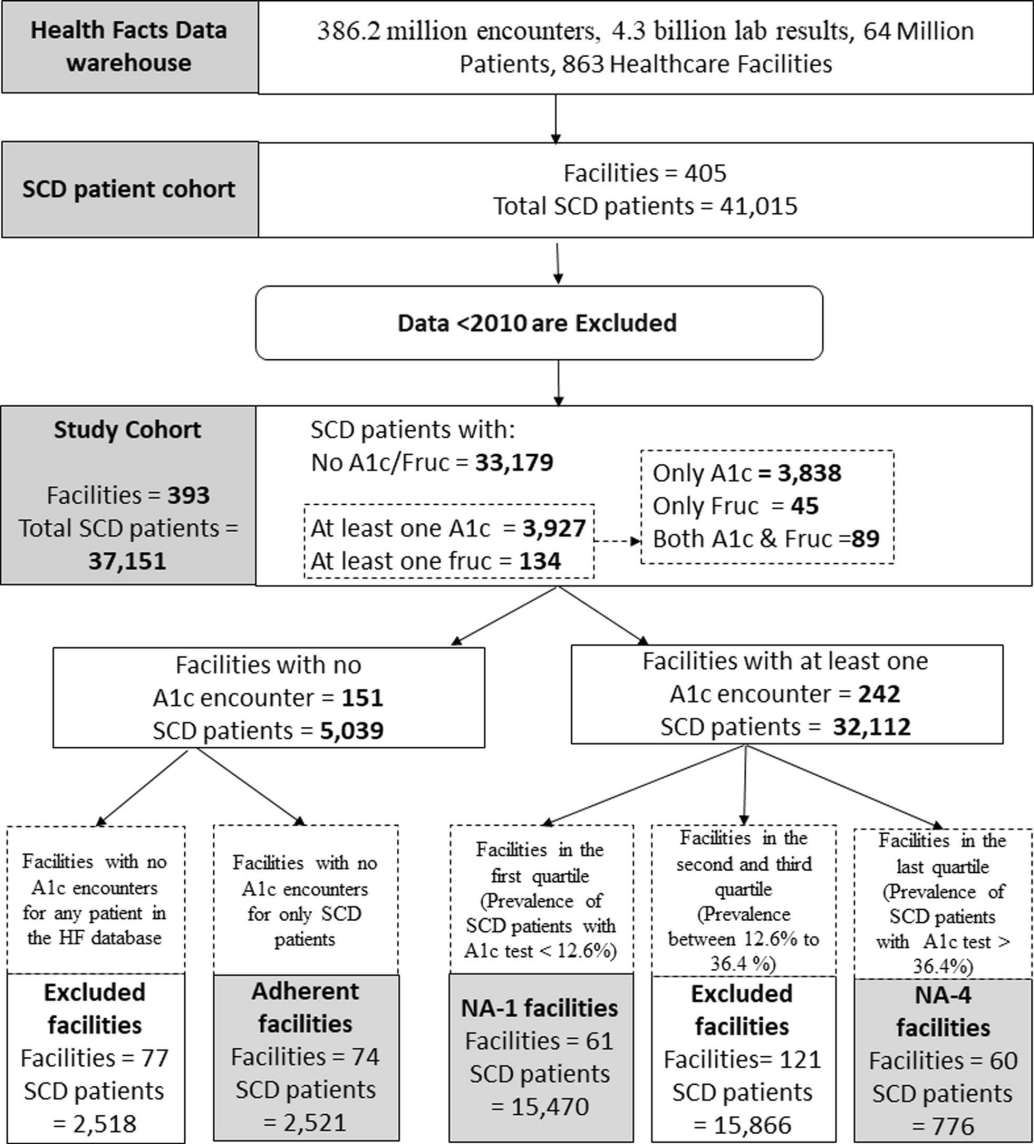
Analysis plan and baseline measure of the utilization of A1c and fructosamine tests in the study cohort. HF data indicated that A1c and fructosamine test orders are not mutually exclusive because 89 patients had both tests ordered. Seventy-seven facilities were eliminated from the HF analysis because there was no evidence that A1c testing was offered within these settings to any patient

The overall study cohort included 393 facilities (with 37,151 SCD patients) out of the 863 facilities that contributed data to HF (Fig. 1). Of the 393 facilities, 151 facilities (5039 patients) had no A1c test orders for patients classified as having SCD while the remaining 242 facilities (32,112 patients) had at least one A1c test order for an SCD patient. From the 151 facilities with no A1c encounters for SCD patients, 77 facilities (2518 patients) were excluded from the analysis because there was no record of an A1c encounter for any patient regardless of SCD diagnosis. The 77 facilities may not perform A1c tests in house or another factor may have limited the inclusion of that data in HF. The remaining 74 facilities (2521 SCD patients) which had no A1c encounters for SCD patients, but performed A1c testing for non-SCD patients, were categorized as “adherent” facilities. Facilities with one or more A1c tests for SCD patients (242 facilities; 32,112 SCD patients) were grouped into quartiles based on the proportion of SCD patients with A1c tests as described in the methods. The first quartile, termed minor non-adherent or NA-1, comprised 61 facilities (15,470 patients). The second and third quartiles comprised 121 facilities (15,866 patients) and are excluded from our analysis. The last quartile, NA-4, comprised 60 facilities (776 patients).

### Overall HF cohort

We did not find encounters in HF for glycated albumin and 1,5 anhydroglucitol tests (LOINC Codes: 13873-5, 1758-2, CPT code: 82985) for SCD patients and focused on fructosamine as the primary alternative test to A1c in this study. Analyzing the HF SCD cohort for A1c and fructosamine orders indicated that 3927 patients (11% of the study cohort) had at least one A1c test and 134 patients had at least one fructosamine test (Fig. 1). There were only 28 facilities from the cohort with fructosamine encounters. One health system with two facilities contributed 76% of the total fructosamine test orders in the HF cohort. Out of 37,151 SCD patients in the cohort, 89 patients had both an A1c and fructosamine test while 3838 patients had only an A1c test and no fructosamine test, 45 patients had at least one fructosamine test and no A1c test and 33,179 patients had no A1c test ordered and no fructosamine test ordered (Fig. 1). Of the 89 patients who had both an A1c and fructosamine test, 12% of A1c tests (63 of 533 A1c tests) from 42 patients were ordered with the fructosamine test during the same encounter.

### Facility characteristics

Facility level characteristics such as the average number of SCD patients in a facility, census region, size of the facility, teaching status, urban and acute care facilities were compared among the HF cohort and the three cohorts (adherent, NA-1 and NA-4) (Table 2). In the HF cohort, there were 134 facilities with a bed size of less than 5 (likely ambulatory), 105 facilities with a bed size between 5 and 100, 133 facilities with a bed size between 100 and 500, and 21 facilities with more than 500 beds. The NA-4 subcohort had more facilities with bed size less than 5 (27 facilities; 44%) compared to NA-1 subcohort (14 facilities; 23%). NA-1 facilities when compared to NA-4 facilities had a higher percentage of teaching facilities (28 facilities; 46% vs 13 facilities; 21%) and acute care facilities (51 facilities; 84% vs 41 facilities; 67%).

**Table 2:** Characteristics of Facilities, Serving Sickle Cell Disease (SCD) Patients, Cerner Health Facts™, 2010–2016

Facility characteristics	HF cohort n = 393 facilities	Adherent (ratio = 0) N = 74 facilities	NA-1 (0 < ratio < 0.126) N = 61 facilities	NA-4 (ratio > 0.363) N = 60 facilities
SCD patients: Mean (Range)*	110 (1–2803)	34 (1–1661)	254 (8–2003)	13 (1–147)
Prevalence of SCD patients with A1c test: Mean % (Range)**	11 (0–100)	0 (0–0)	7 (0.2–12.5)	67 (36.5–100)
Census region: No. of facilities (%)				
Midwest**	79 (20)	19 (26)	7 (11)	13 (21)
Northeast*	68 (17)	9 (12)	7 (11)	17 (28)
South**	162 (41)	23 (31)	39 (64)	9 (15)
West*	84 (21)	23 (31)	8 (13)	21 (35)
Bed size range: No. of facilities (%)				
< 5*	134 (34)	20 (27)	14 (23)	27 (44)
5–99**	105 (27)	39 (53)	12 (20)	18 (30)
100–199*	59 (15)	7 (10)	12(20)	6 (10)
200–299**	45 (12)	3 (4)	15 (26)	6 (10)
300–499**	29 (7)	1 (1)	6 (10)	1 (2)
500+	21 (5)	4 (5)	1 (1)	2 (3)
Teaching: No. of facilities (%)**	109 (28)	14 (19)	28 (46)	13 (21)
Urban: No. of facilities (%)	300 (76)	55 (74)	48 (79)	48 (79)
Acute care: No. of facilities (%)	286 (73)	59 (80)	51 (84)	41 (67)

Ratio refers to the prevalence of SCD patients with A1c tests

SCD, Sickle Cell Disease

Adherent facilities are facilities with at least one patient with an SCD diagnosis code but no code for an A1c test ordered for an SCD patient

Minor non-adherent (NA-1) facilities are facilities where the prevalence of SCD patients with A1c tests is lesser than the 25th percentile value (12.6%)

Major non-adherent (NA-4) facilities are facilities where the prevalence of SCD patients with A1c tests is greater than the 75th percentile value (36.3%)

Statistical significance compared between Adherent, NA-1 and NA-4 cohorts are indicated by *p < 0.05 and **p < 0.001

### Adherent, NA-1 and NA-4 facility cohorts

Patient level characteristics such as percentage of A1c utilization, age, sex, race, and sickle cell diagnosis groups were compared among the baseline HF cohort and the three sub-cohorts (Adherent, NA-1 and NA-4) (Table 3). We observed that patients tended to be younger in the NA-1 than in NA-4 group (Mean age, 24.5 years vs 51.2 years).

**Table 3:** Characteristics of Sickle Cell Disease (SCD) patients, by facility, level of A1c testing, Cerner Health Facts™, 2010–2016

Baseline characteristics	No. of sickle cell disease patients (%)^a^			
	HF cohort N = 37,151	Adherent N = 2521	NA-1 N = 15,470	NA-4 N = 776
SCD patients with A1c test**	3927 (11)	0	841 (5)	446 (57)
Gender				
Male	16,235 (44)	1119 (44)	7257 (47)	332 (43)
Female	20,822 (56)	1363 (54)	8193 (53)	444 (57)
Age in years: mean (range)*	30.8 (0–90)	38.6 (1–86)	24.5 (6–46)	51.2 (5–84)
Race				
African American**	23,764 (64)	2052 (81)	12,366 (80)	354 (46)
Asian/Pacific Islander*	401 (1)	16 (0.6)	138 (0.9)	9 (1)
Biracial*	70 (0.2)	1 (0.04)	49 (0.3)	4 (0.5)
Caucasian**	8933 (24)	290 (12)	1147 (7)	301 (39)
Hispanic	169 (0.4)	5 (0.2)	62 (0.4)	6 (0.7)
Native American*	78 (0.2)	5 (0.2)	15 (0.1)	4 (0.5)
Other*	3736 (10)	152 (6)	1693 (11)	98 (13)
Diagnosis groups				
SC-Thal (WoC)*	850 (2)	59 (2)	454 (3)	12 (2)
SC-Thal (WC)	298 (0.1)	20 (1)	80 (0.5)	4 (0.5)
HbSS (u)**	14,712 (40)	1271 (50)	5565 (36)	262 (34)
HbSS (WoC)**	4914 (14)	414 (16)	4201 (34)	218 (28)
HbSS (WC)**	7255 (20)	524 (21)	3679 (24)	224 (29)
HbSC (WoC)*	1194 (3)	61 (2)	810 (5)	7 (1)
HbSC (WC)*	217 (0.6)	7 (0.3)	113 (0.7)	1 (0.1)
Other SC (WoC)**	311 (1)	67 (3)	158 (1)	5 (0.6)
Other SC (WC)**	6874 (19)	91 (4)	225 (1)	15 (2)

SC, sickle cell; Thal, thalassemia; WoC, without crisis; WC, with crisis; u, unspecified

Statistical significance between Adherent, NA-1 and NA-4 cohorts is indicated by *p < 0.05 and **p < 0.001

^a^Subgroups total to less than 100% of the total cohort due to missing data

**Fig. 2:**
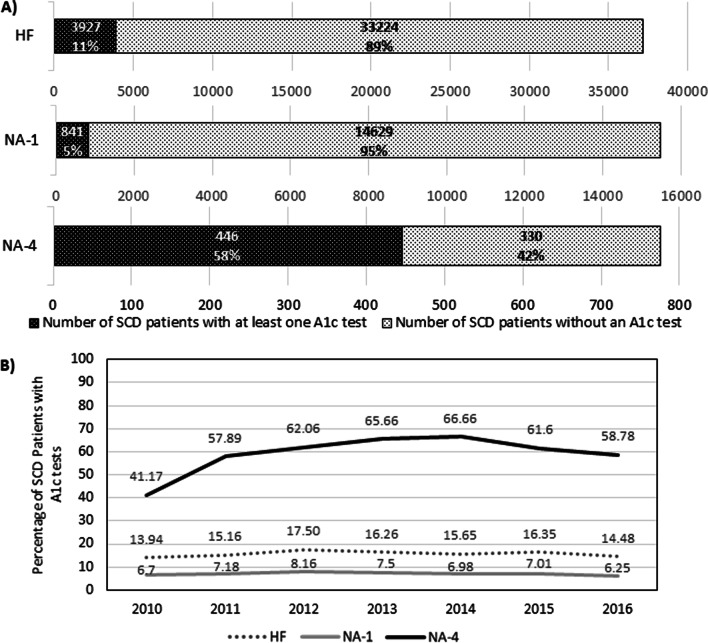
**a** Sickle-cell patients with A1c encounters in the HF cohort, Minor non-adherent (NA-1) and Major non-adherent (NA-4) facilities. **b** Percentage of sickle cell patients with A1c encounters per year from 2010 to 2016 in HF, Minor non-adherent (NA-1) and Major non-adherent (NA-4) facilities

Only 5% of the SCD patients at the NA-1 facilities (841/15470 patients) received at least one A1c test, while 58% of the SCD patients in the NA-4 facilities (446/776 patients) had at least one A1c test (Fig. 2a). Evaluating the annual frequency of A1c encounters shows that the SCD patients with A1c tests at the NA-4 was consistently higher every year between 2010 and 2016 when compared to the NA-1 (Fig. 2b).

## Discussion

We report mis-utilization of A1c testing and apparent underutilization of fructosamine testing for the SCD population from diverse facilities (acute and non-acute, teaching and non-teaching, urban and rural, small ambulatory to large facilities) across the major census regions of the US. Among facilities with known A1c testing (316 facilities), fewer than 25% (74 Adherent facilities) demonstrated full adoption of the SCD testing guidelines, with the lack of adoption most notable among the facilities in the NA-4 subcohort (Fig. 1).

The data suggest lower compliance at ambulatory facilities (the category of facilities with 0–5 beds) and non-teaching facilities. One possibility for this observation is that providers in ambulatory facilities are more apt to order A1c for SCD patients because of greater familiarity with this test or less familiarity with treating patients with SCD. There may be a delay in awareness in non-teaching facilities about guidance from professional groups such as the Joint Commission about the limitations of A1c for the SCD population [29]. Providers may also order A1c tests because they are incented to attain goals through the Centers for Medicare and Medicaid Services Pay for Performance program [30]. As of 2021, these goals do not take into consideration those with SCD and other hemoglobinopathies. If this is a contributing factor to mis-utilization of A1c testing, these goals may need review and modification.

Based on the data, NA-1 facilities serve younger SCD patients and consist of a higher proportion of pediatric facilities than NA-4 where the A1c or fructosamine test is less likely to be ordered for monitoring chronic diabetic mellitus. The ADA Standards of Care use A1c values but the caveat about the use of A1c testing in people with hemoglobinopathies, independent of age group, is not emphasized [31].

Several possibilities exist for the overall low utilization of fructosamine. Because the majority of fructosamine tests identified were within a single health system, it is possible that HF did not receive fructosamine utilization data from all contributors, for example if they were sendout orders. Another explanation is that other tests may have been used, such as glucose screening and monitoring, in making diagnostic and treatment management decisions. This may be preferred because standardized cut-offs have not been established for fructosamine testing and must be determined and validated by each laboratory offering the test. The limitation of glucose screening is that it provides the status of glucose levels at the time of the test and not over a period of time. Additional studies are needed to review the use of glucose testing within a sickle cell populationWe noted co-ordering of A1c and fructosamine testing for sixty-three SCD patients, potentially suggesting an effort to gain greater familiarity with the fructosamine test or compliance with local guidelines. A follow up study to better understand why A1c is ordered may provide insights to changing clinician ordering behavior. Collectively this analysis shows that inappropriate A1c orders and underutilization of fructosamine test orders for SCD patients are quality gaps. From a public health perspective, this may translate to increased number of patients not receiving optimal care and requiring additional health resources. This suggests the need for a follow up study that examines the cost and burden of inappropriate A1c testing within the underserved SCD population.

Several interventions may be considered to address this quality gap. Potential interventions may include clinician education about the appropriate utilization of A1c and fructosamine testing, facilitated using professional guidance from National Institute of Diabetes and Digestive and Kidney Diseases and the Centers for Disease Control and Prevention [6, 22]. It is worth exploring the usefulness of integrating clinical decision support within EHRs to intercept A1c tests ordered for sickle cell patients and recommend more appropriate testing modalities. Other communication and education approaches (e.g., grand rounds, CME course offerings, communication campaign) targeted to reducing mis-utilization of A1c testing are also warranted.

### Limitations

This study has limitations. First, analyzing de-identified EHR data is challenging because there is no way to verify the accurate use of diagnostic codes (ICD-9-CM, ICD-10-CM) in HF [32, 33]. Variations exist in the coding and accuracy of both sickle cell disease overall and the specific sickle cell genotypes (e.g., HbSS, HbSC, sickle cell thalassemia) [34]. All variants were treated consistently in this analysis; the accuracy of the sickle cell genotype is unlikely to affect our analyses as any variant of sickle cell genotypes may influence the reliability of A1c analysis [35–38]. Previous assessment of the accuracy of SCD classifications based on diagnostic codes indicates that despite high sensitivity and predictive value positive, specificity might be lower when classification of SCD is based on a the ocurrence of at least one (as opposed to two or more) ICD codes for a patient [39]. We cannot rule out the possibility that some of our SCD cases were false-positive classifications but do not expect false-positives to alter our finding regarding mis-utilization of A1c testing in the sickle cell population. Additionally, as our analysis was focused on potential associations to the least and most adherent subcohort, we did not include the second and third quartile facilities (NA-1 nor NA-4) despite a significant number of patients. Another possible limitation is that some A1c tests may not be included in HF due to the absence of the Cerner laboratory module at some sites or the use of point-of-care A1c testing that does not populate the pathology database tables [40]. Also, the same concerns may apply to our observation of limited ordering of fructosamine and the absence of other alternative tests.

## Conclusion

Analysis of data abstracted from HF provided evidence for mis-utilization of A1c and under-utilization of alternate testing in a sickle cell population across facilities. Quality improvement initiatives to improve compliance with professional guidance to promote the use of alternate tests, such as fructosamine, in a sickle cell population can support the accurate and timely the diagnosis and management of diabetes in the sickle cell population. This work serves as an important example of the value of aggregate EHR data in research that can inform care for underserved populations. The informatics strategy described can be applied to many other large scale questions related to laboratory test utilization.

## Data Availability

The datasets used and/or analysed during the current study are available from the corresponding author on reasonable request.

## Abbreviations

A1c: Glycated Hemoglobin
EHR: Electronic Health Record
HF: Health Facts
NA-1: Minor non-adherent
NA-4: Major non-adherent
ICD-9-CM and ICD-10-CM: International Classification of Diseases, Ninth Revision and Tenth Revision, Clinical Modification codes
